# Report on Proceedings of the Tenth Annual European CME Forum, Dublin, Ireland, November 2017

**DOI:** 10.1080/21614083.2017.1421376

**Published:** 2018-01-10

**Authors:** Ron Murray

**Affiliations:** ^a^ Independent CME/CPD Consultant, Pickering, UK

**Keywords:** Quality, collaboration, engagement, outcomes, partnership, stakeholders

## Abstract

The august setting of the Royal College of Physicians of Ireland saw participants from 17 different countries assemble for the Tenth European CME Forum between 8th and 10th November 2017. The main themes of the meeting were classified under the headings Inward (Educational design), Outward (Listening to others) and Onward (Collaborations and partnerships) addressed via a combination of presentations, interactive workshops, posters, and panel discussions. Topics explored included team engagement, the voice of the patient, harmonisation in European accreditation, competencies for CME professionals, and publishing in CME. Discussion evoked both consensus and contention and provided participants with excellent networking opportunities moving forward to the next decade of Forum meetings.

Portraits of some of Ireland’s most venerable physicians e.g. Robert Graves, William Stokes, John Cheyne, and Sir Henry Marsh on the walls of the meeting rooms of the Royal College of Physicians of Ireland (RCPI) provided a suitably erudite backdrop for the 10th European CME Forum on 8–10th November 2017 in Dublin. The forum adopted a thematic approach under the headings:

- INWARD – dealing with theory and practice in educational design
- OUTWARD – consideration of the views of the range of stakeholders in CME/CPD
- ONWARD – discussion of existing and potential collaborations and partnerships

Building on preferences elicited from the pre-meeting needs assessment, the themes were addressed via a combination of presentations, interactive workshops and panel discussions that fully engaged the attendees who represented 17 different countries.

Day 1 began with an introductory welcome from Professor Mary Horgan, president of RCPI and Professor Hilary Hoey, Director of Professional Competence at RCPI who both emphasised the College’s recognition of the importance and effectiveness of CME/CPD within the framework of the history of RCPI since its founding in 1654.

For the opening session, under the INWARD banner, Don Moore of Vanderbilt University provided a series of tips on approaching educational design in CME from a project management perspective. The nugget from his presentation was to “start with the end in mind” and organise learning for transfer by incorporating the ADDIE model (Analyse, Design, Develop, Implement and Evaluate) A comprehensive summary diagram for this approach is shown in Figure 1.

**Figure 1.**
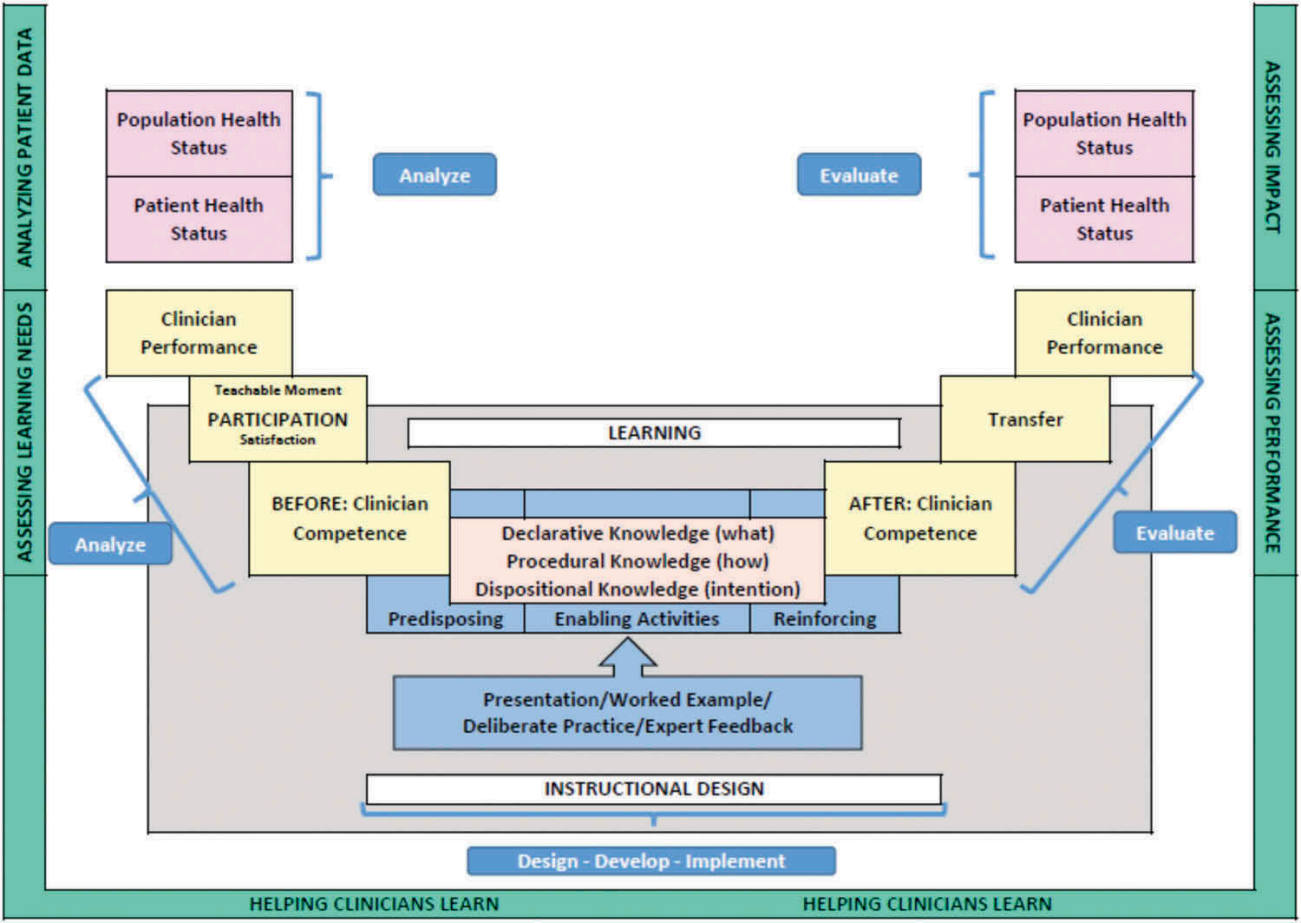
Incorporating the ADDIE model into CME design (with permission from Don Moore Ph.D).

Day 1 concluded with four concurrent workshops continuing the INWARD theme. These workshops were facilitated by delegates from European and USA-based organisations as follows.

## Workshop 1A: enhancing CPD: quality improvement and best practice

**Figure 2.**
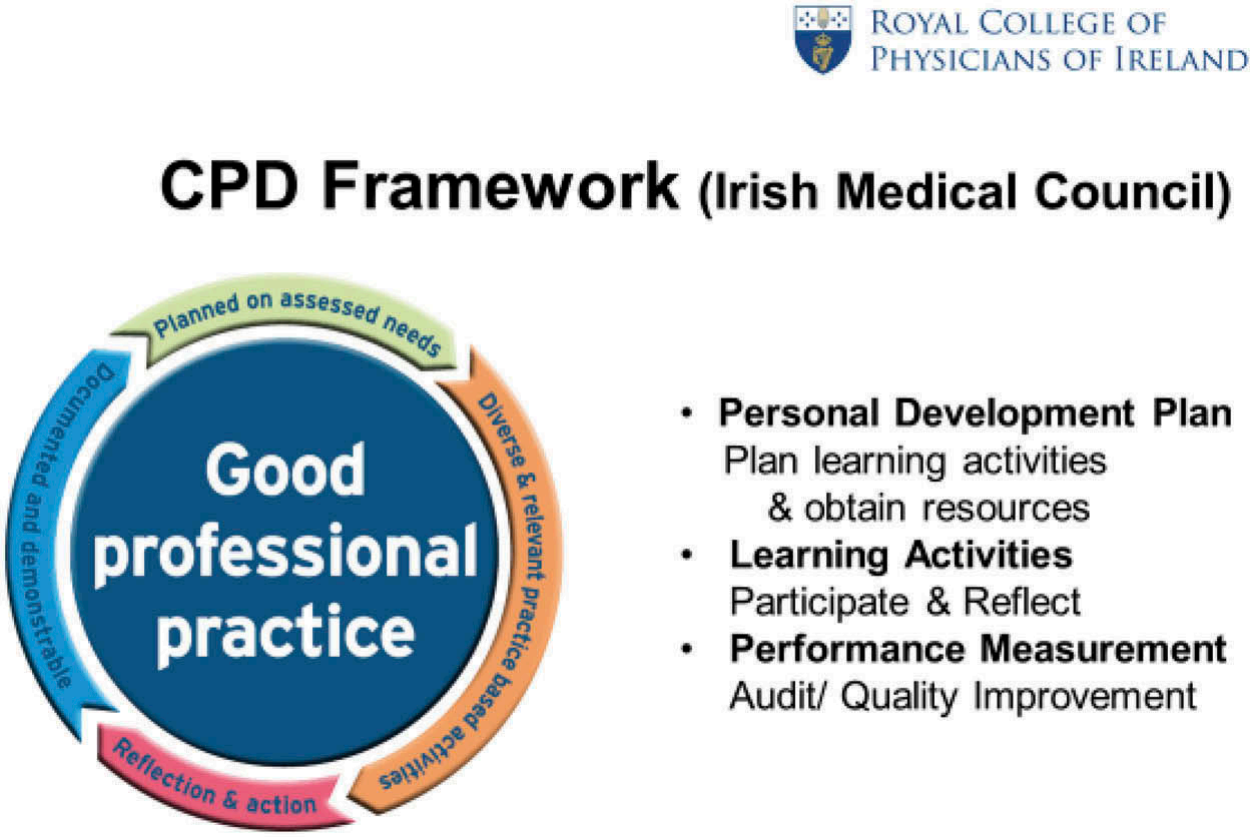
A framework for CME/CPD in Ireland (with permission from Professor Hilary Hoey).

A delegation from RCPI outlined the procedures involved in the maintenance and recording of CPD in Ireland which has been a legal requirement since May 2011. They highlighted the changing face of healthcare and the need for partnerships among health professionals, educators, accreditors, regulators and employers to achieve the desired aim of optimal patient care. The framework for administering CPD in Ireland is outlined in Figure 2 and it was encouraging to learn of the steady progress being made with increased enrolment and increased numbers of physicians meeting the CPD requirements.

Promotion, support and investment in the programme were cited as key elements to continue the progress being made. Workshop participants completed an exercise to provide suggestions for appropriate design and implementation of effective learning activities by considering questions such as:

- How do we enhance participation and engage the non-engagers?
- How do we make CPD relevant to participants?
- Does completing a Personal Development Plan improve Knowledge and Performance?
- How do we measure the effectiveness of a learning activity?
- Are Patient Feedback and Peer Review effective tools for Performance Appraisal?
- How do we measure practice outcomes e.g. Quality Improvement and Clinical Audit?
- Who should monitor compliance?
- What actions should be taken in cases of non-compliance?

## Workshop 1B: building the case for self-aware learners and effective education

Kate Regnier, Executive Vice President of the Accreditation Council for Continuing Medical Education (ACCME) provided a very comprehensive workshop that addressed issues such as

- The need to break barriers between formal and informal learning
- Differences in teaching students, residents, fellows, and practising physicians, for example
  - Medical students: need fundamental skills
  - Residents: have some skills and experience, but are open to more
  - Students and residents take at face value that they are still learning
  - Professionals in practice: make a lot of incorrect assumptions and may have errors in assumed knowledge and self confidence

She also emphasised the growing awareness of the professionalism of CME educators noting that, in the not too distant past, CME often meant getting a faculty expert to deliver a lecture. Experts were regarded as the keepers of knowledge, and CME was about knowledge transfer with faculty having no real pressure or obligation to be good educators. However, in the modern era, with immediate access to information, knowledge transfer is no longer the currency of CME, but the emphasis is on skill development and communication. The role of CME/CPD educators is to develop these skills and partner with accreditors whose role is more “coach” than “cop” in nurturing the skills of CPD professionals. Certain challenges faced in educating practising physicians were identified such as their wishing not to be confronted with what they perceive as “basic” information, even though information may have been updated, and they may be relying on incorrect and/or outdated information. A remedy of sorts was suggested i.e. naming something a “Mastery Programme” to overcome psychological barriers to learning or re-learning basic information.

**Figure 3.**
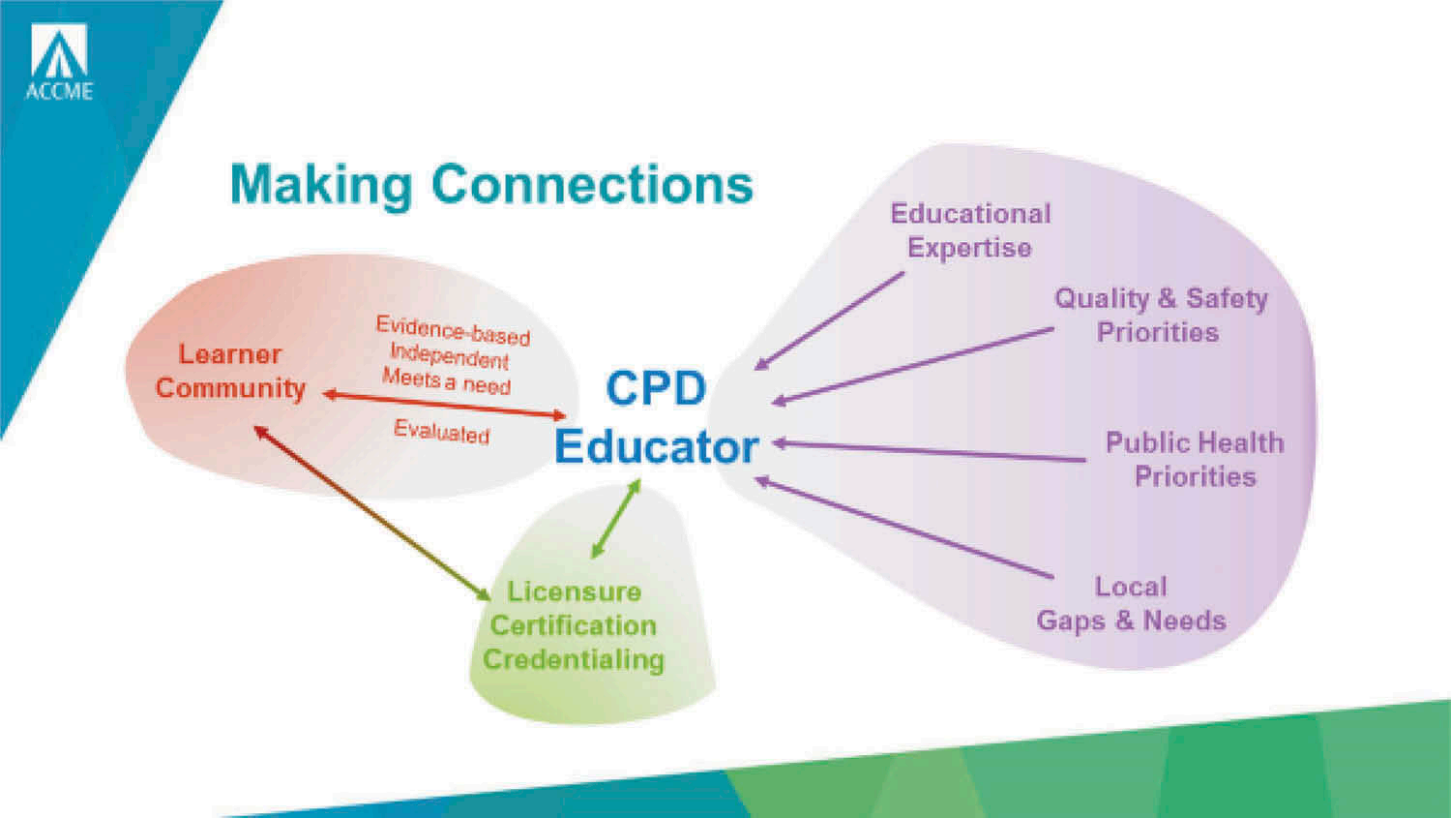
The central role of the CPD Educator (with permission from Kate Regnier).

The central role of the CPD professional was then considered in encouraging mastery learning and self-reflection so that the evolution of CME can be a partnership between creative educators and insightful learners. This central role for the professional CPD educator is illustrated in Figure 3.

## Workshop 1C: competencies for CME professionals

**Figure 4.**
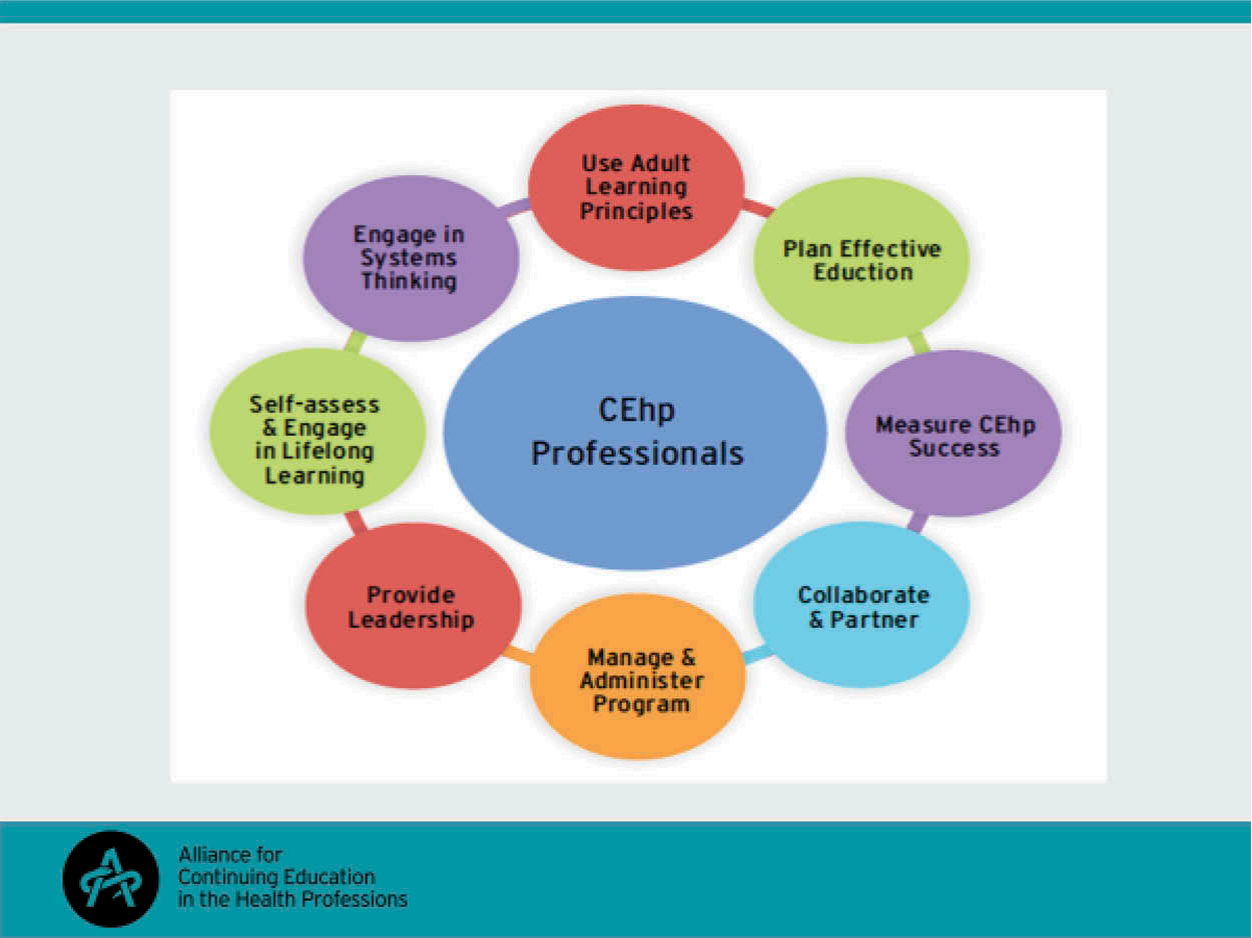
ACEhp’s eight Competency Areas.

Gayla Bruner and Steven Kawczak, respectively current and incoming president of the Alliance for Continuing Education in the Health Professions (ACEhp) outlined their organisation’s mission of connecting healthcare education professionals to promote best practices that improve patient care and its vision of advancing continuing health education for healthcare professionals. A central tenet of the Alliance’s approach has been to provide a framework for job descriptions, performance expectations and career growth for healthcare education professionals. To this end a set of competency areas and specific learning competencies within each area have been developed over recent years and promulgated as necessary skills for lifelong learning as a healthcare educator. The competency areas were outlined as shown in Figure 4 and a range of competencies subsumed within these areas was described as they would apply in a professional’s journey from novice to expert. Participants were given the opportunity to design an educational activity based on a case-based scenario provided and report on the competencies utilised in the design plan.

The main benefits of using the competency model were listed as:

- Providing a template for planning
- Professional development
- Staffing & Recruiting
- Being the “gold standard” for a CE professional.

## Workshop 1D: lifelong learning in Europe: opportunities and challenges in achieving quality

Representatives of industry who had contributed to a recent article published in the Journal for European CME (1) asked participants to consider:

- the different perspectives of the definition of quality in CME
- the role of various stakeholders (the pharmaceutical industry in particular) in ensuring quality
- opportunities for future alignment and cooperation in consistently achieving high-quality learning programmes

It was mooted that the provision of CME in Europe is somewhat fragmented with providers including Universities, Physician Associations/Societies, Medical Education/Communication Companies, and Pharmaceutical Companies although perhaps united by a common goal for optimal standards of care. With various quality criteria for education in use by accrediting bodies, a pharmaceutical industry special industry group affiliated with the Global Alliance for Medical Education (GAME) the International Pharmaceutical Alliance for Medical Education (iPACME) has started to discuss a common set of quality principles for Europe. These discussions led to a suggested consensus on medical education quality principles for the industry comprising:

- Ethical, transparent and responsible engagement
- Needs-based, up-to-date, balanced and objective content
- Robust and standardised processes for the delivery of educational programmes

The rationale for this suggestion that an industry standard on quality principles is important for Europe was based on the fact that the pharmaceutical industry is already involved in providing support to third-party-provided medical education, collaborative partnerships, industry-provided professional development and medical disease education. Furthermore, the workshop leaders felt that the industry is seen to have:

- expertise in clinical development, disease areas, healthcare systems and educational science.
- product information that is rigorously reviewed to be accurate, fair and objective.
- strict compliance regulations.
- mandatory training of employees, audit processes and governances provided by compliance.
- dedicated departments for medical education, specialised functions for the provision of grants.

**Figure 5.**
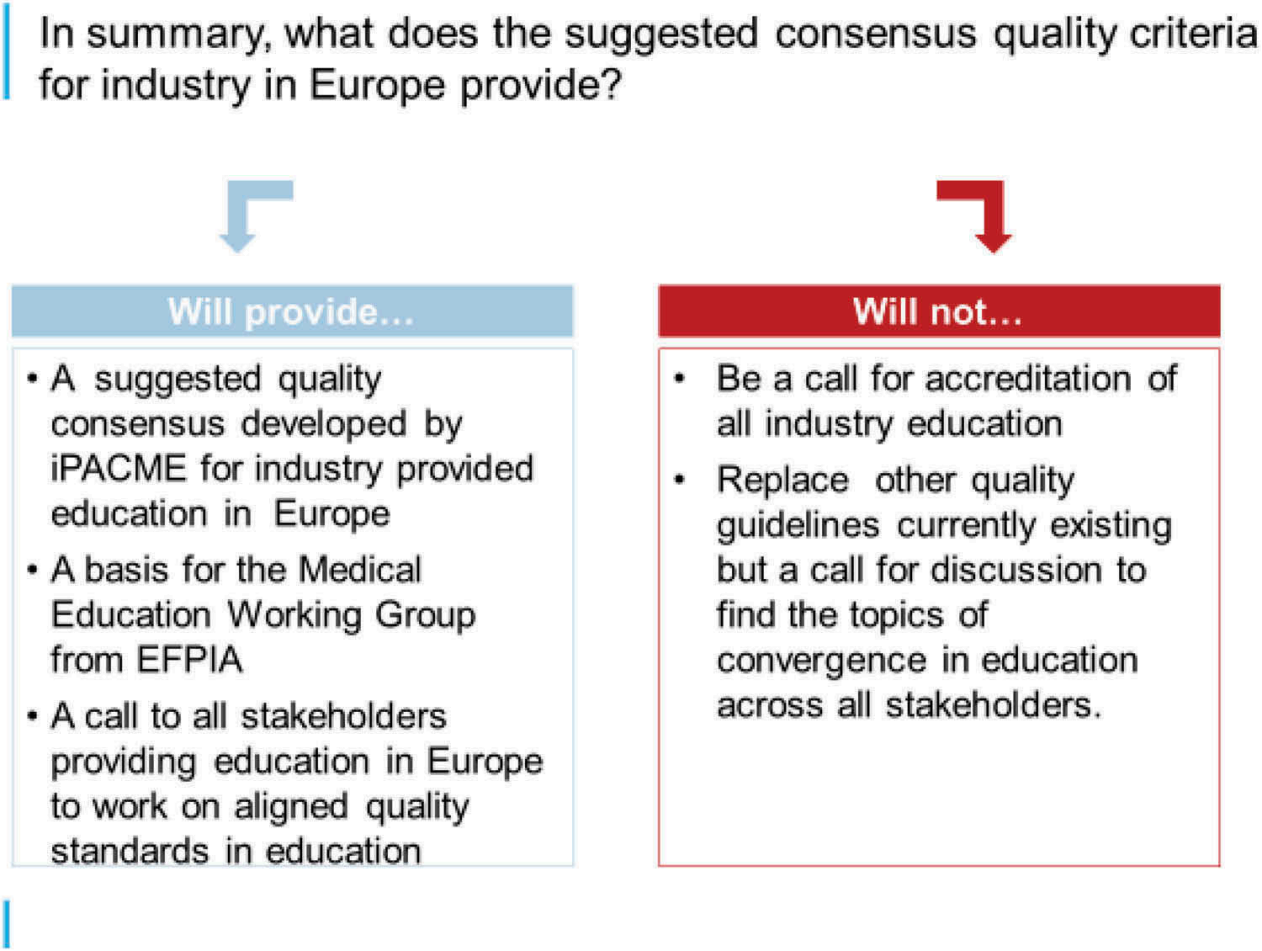
A proposal for industry-based medical education quality criteria in Europe.

They also contended that active collaborations between pharmaceutical companies and medical societies and scientific experts tend to demonstrate high integrity, clear roles & responsibilities. The suggested consensus is summarised in Figure 5.

**Figure 6.**
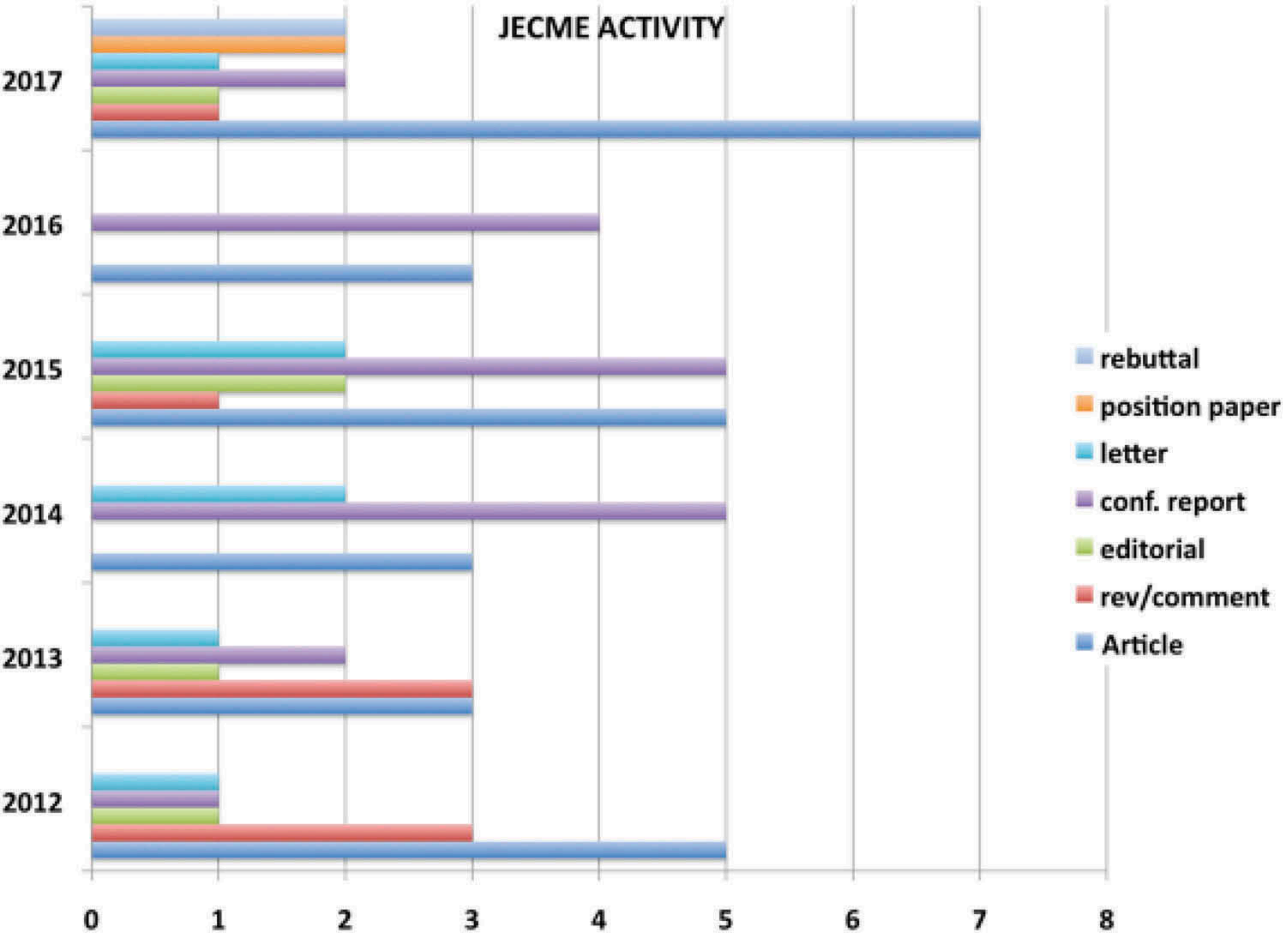
Journal of European CME content 2012–2017.

Day 2 opened with a review of the Journal of European CME (www.jecme.eu) by Editor-in-Chief Robin Stevenson from its inception in 2012 to 2017 with current publishers Taylor & Francis. Prof. Stevenson described the journal’s activity in terms of content as shown in Figure 6 and urged attendees to provide further content across the spectrum of headings indicated. The fact that the journal is open-access provides a much quicker editorial and acceptance time for submissions.

A reflection and review session followed to allow all participants to share in the highlights from the previous day’s workshops and the main point that emerged was that CME professionals and learners need to be more aligned and that partnerships need to be forged among the various stakeholders in the CME/CPD enterprise to support the steady move away from an information dissemination model.

The OUTWARD theme of “listening to others” was introduced by Dr Graham McMahon, President and CEO of ACCME on a return visit to his native Ireland. The theme was exemplified through a presentation by Carolin Sehlbach of Maastricht University of preliminary results of an international study on national recertification systems in Europe that she has conducted as part of her Ph. D studies.

**Figure 7.**
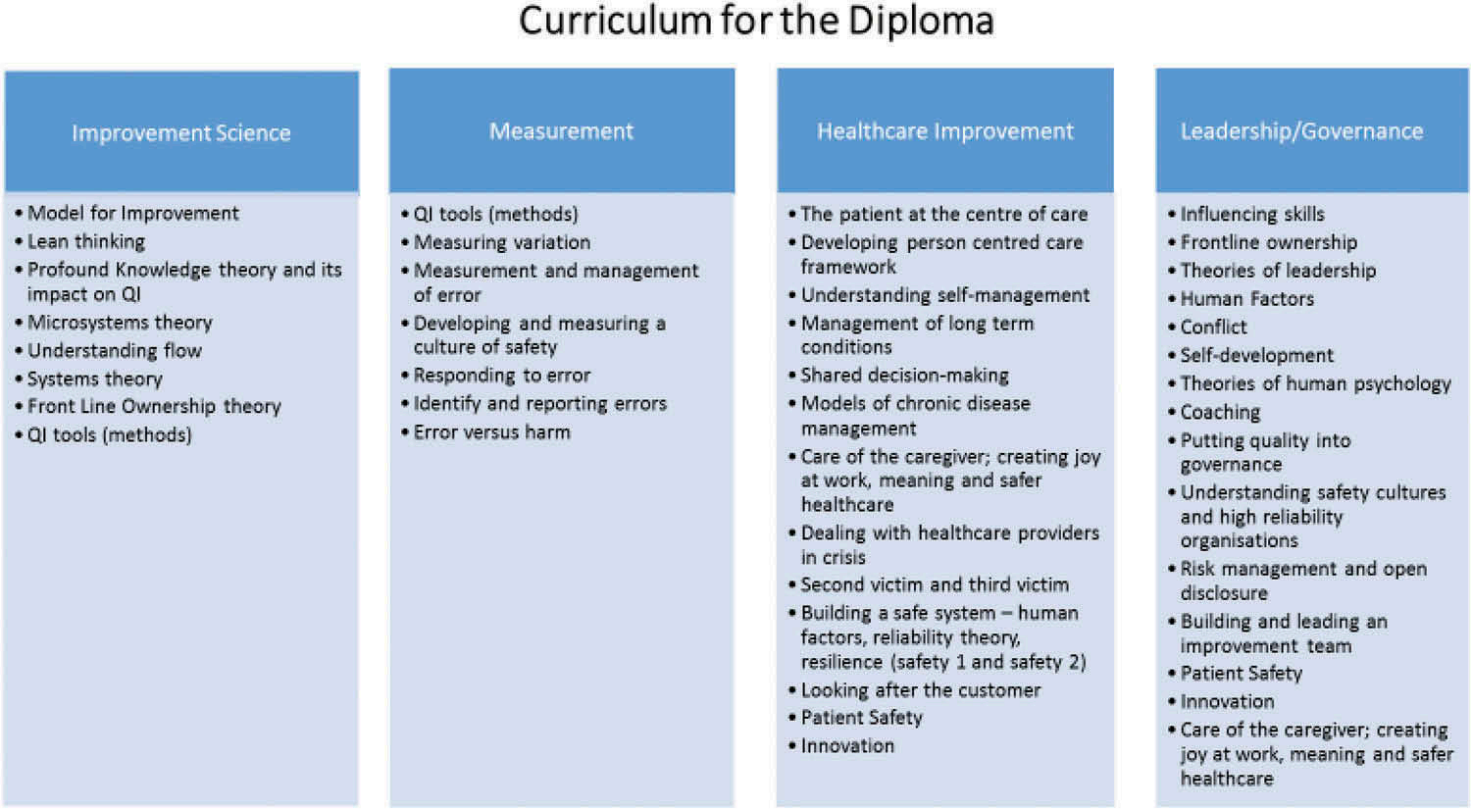
Irish diploma in leadership and quality in healthcare.

The OUTWARD workshops completed the morning session of Day 2.

## Workshop 2A: engaging the team

A delegation from RCPI described the Diploma in Leadership and Quality in Healthcare programme that has been established by the Health Service Executive (HSE) of Ireland and RCPI. The aim of the programme is to build leadership skills and expertise in quality improvement (QI) and to develop a common language between administrators and clinicians to achieve a common goal of improving patient care. The main points emerging from this programme are that:

- QI can identify practice gaps at community health/population health level and assess whether gap is closed by the QI programme.
- a challenge exists if CME only targets physicians, when an entire team is essential for quality improvement.
- Partnerships can be forged between QI and CPD in the workplace
  - QI concepts, principles, skills can be generalisable to CME/CPD
- Future strategic directions for CME make it
  - no longer acceptable for physicians to receive education in their silo
  - necessary for the team to be considered as the “education unit”.

An outline of the curriculum for the Diploma programme is shown in Figure 7.

Workshop 2B: The voice of the patient: how to fill the communication-gap between doctors and patients

**Figure 8.**
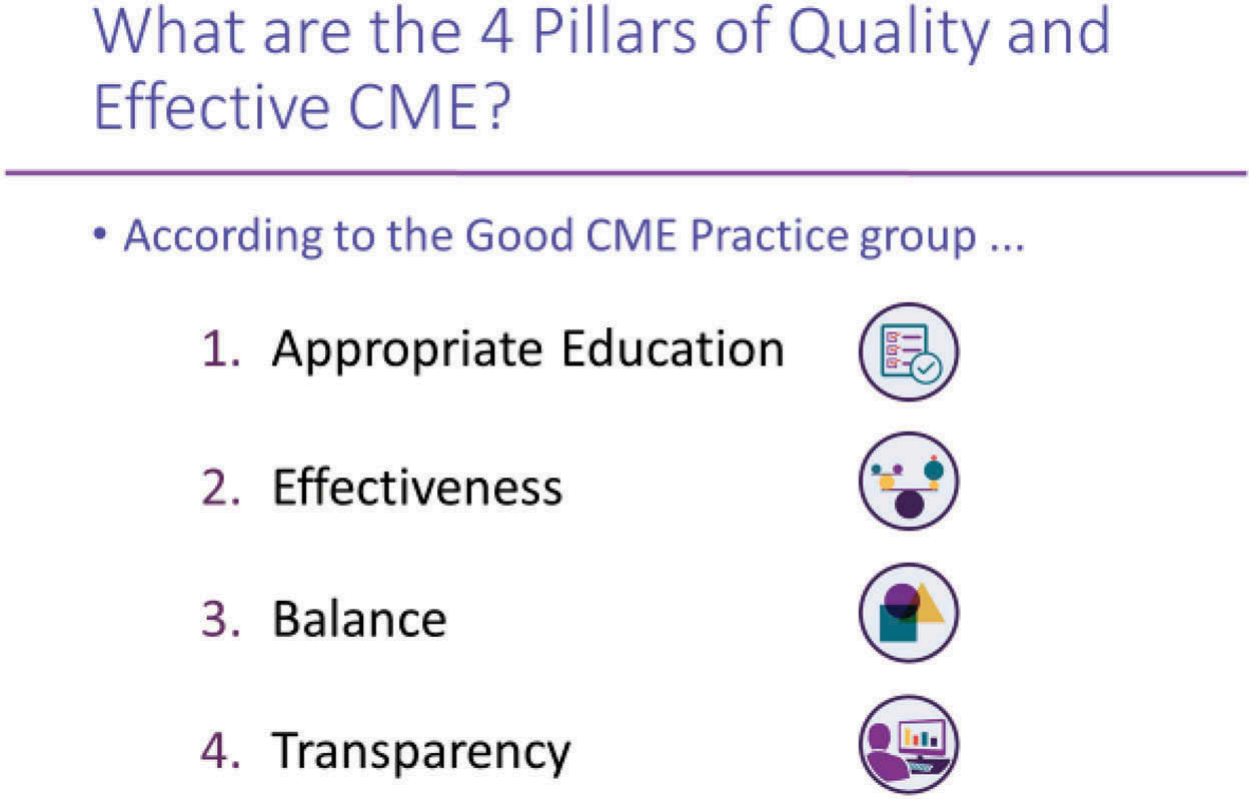
The good CME practice group’s four pillars.

Two representatives of Lupus Europe (www.lupus-europe.org) which is an umbrella association of 26 national lupus self-help organisations throughout Europe, provided a compelling patient perspective from this very engaged patient advocacy group representing around 10% of the European Lupus patient population. Some of their achievements include the creation of ERN ReConnect a European reference network for connective tissue disorders and the harmonisation of lupus guidelines across the EU.

Much of the discussion centred on communication gaps between doctor and patient with specific reference to adherence:

- 24% of patients with lupus do not adhere to treatment recommendations.
- a problem exists in enabling health professionals to support adherence without taking on a “parenting” role.

The ideal solution would involve active listening and collaborative solutions but the reality

of time restraints, reliance on patient education materials (handouts), and lack of care continuity mitigate against success.

Examples of more effective communication techniques include:

- teach back: clinician asks patient to repeat back what she has just heard
- encourage shared decision-making (SDM), but understand that some patients want to be “told” what to do.
- incorporate tools such as the Lupus App: patients provide journal entries about what is happening in their lives, the app picks up on key words that may require clinical attention.
- prompt the patients before the appointment to come prepared to discuss their 3 most pressing concerns.
- motivate clinicians to improve their communication skills by showing evidence supporting the links between SDM and improved patient outcomes.

## Workshop 2C: future of CME accreditation in Europe: harmonisation through dialogue and consensus

This workshop chronicled the emergence of a recently formed organisation, the CME-European Accreditors Association (CME-EA) a not-for-profit association of independent European accreditors, also open to CME accreditation entities on the national level. (see www.cme-ea.eu)

CME-EA was founded to address a gap in the European CME/CPD environment, there being no umbrella organisation in Europe to oversee international healthcare systems. CME-EA considers that the lack of an official government mandate creates significant challenges to implementing international principles and rules to facilitate physician mobility without bureaucratic barriers. This could allow CME certificates that would be acceptable to both European accreditors and national regulators.

Therefore, the new association has committed to work towards the harmonisation of CME/CPD accreditation through dialogue and consensus.

CME-EA’s mission is to promote harmonisation of CME/CPD accreditation as part of a quality assurance process aiming to improve physicians’ performance and patient outcomes. The organisation supports the shared principles of international accreditation systems that ensure:

- learning activities are developed to address the needs and professional practice gaps of members of the target audience.
- the content is informed by evidence and bias is minimised.
- learning activities are designed to provide maximum educational impact.
- learning activities are planned and managed to ensure independence from external interests.
- there is rigorous evaluation of educational outcomes at levels including knowledge, competence, performance, and health outcomes.
- accreditation standards are consistently and fairly applied and continuously enhanced.
- rigorous standards for training and evaluation of reviewers will be upheld as part of the accreditation process.
- opportunities for discussion of the principles and procedures in accreditation of CME/CPD will be made available all interested parties.

The take home message from this session was that a growing number of accreditors wish to engage in a process based on an agreed set of Europe-wide principles and rules.

## Workshop 2D: the mountains we climb: challenges for the provider

Members of the good CME Practice Group (http://gcmep.org/) used the group’s four main principles as shown in Figure 8 to provide a framework for participants to answer some questions relating to the challenges of different markets, different specialities, different accreditors and different cultures that affect the provision of CME in Europe.

**Figure 9.**
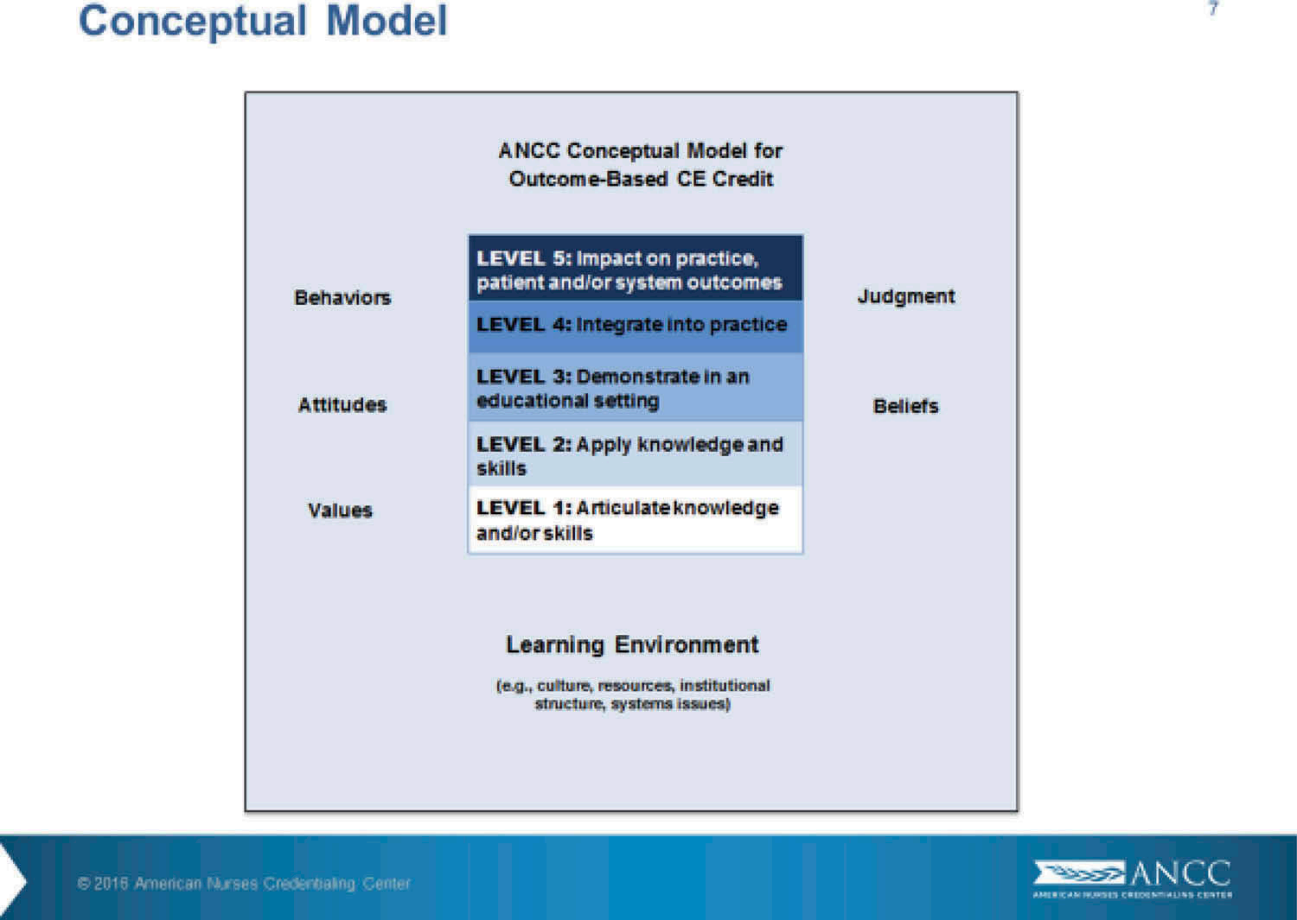
ANCC’s model for competency-based continuing education credit.

The workshop elicited the following group responses for each principle:

appropriate education

- specific local and regional needs must be addressed.
- teaching skills need to be matched with knowledge.

effectiveness

- faculty development cannot be ignored.
- a longitudinal relationship needs to be established with learners.
- data access is a challenge.

balance

- address “real” needs.
- present all options.
- resolve conflicts of interest.

transparency

- role of commercial supporters.
- content validity checked.
- language used – 24 “official and working” in Europe.

A question and answer session with a panel of local healthcare professionals was conducted over lunch and raised some interesting points from the learner’s perspective such as:

“Specialists tend to be retrenched in their attitude to team-based learning”.

“Multidisciplinary courses may be expedient rather than appropriate”.

“Pharma are not interested in supporting nursing education because we are not prescribers”.

“e-learning is futile without assessment”.

“twitter feeds from conferences can be very useful”.

“inter-professional learning is becoming more important”.

“we love being tested!”.

The ONWARD theme commenced with a thought-provoking presentation by Kathy Chappell and Jann Balmer from USA on the progression of CME towards being competency-based rather than time-based. They posed the simple but meaningful question in relation to learning and change- “does time matter?” Participants were asked to consider what should be measured instead and suggested metrics such as competence (defined as the ability to apply knowledge and skills), changes in practice, and impact on practice. A conceptual model (see Figure 9) was provided to illustrate a pilot project being implemented by the American Nurses Credentialing Centre (ANCC). This model comprises five levels ranging from the articulation of knowledge and/or skills to having an impact on practice, patient and/or system outcomes. In this model a Level 1 activity might be conducting a literature review to identify best practices and a Level 5 activity could be the assessment of the impact of a learning programme with partner organisations.

**Figure 10.**
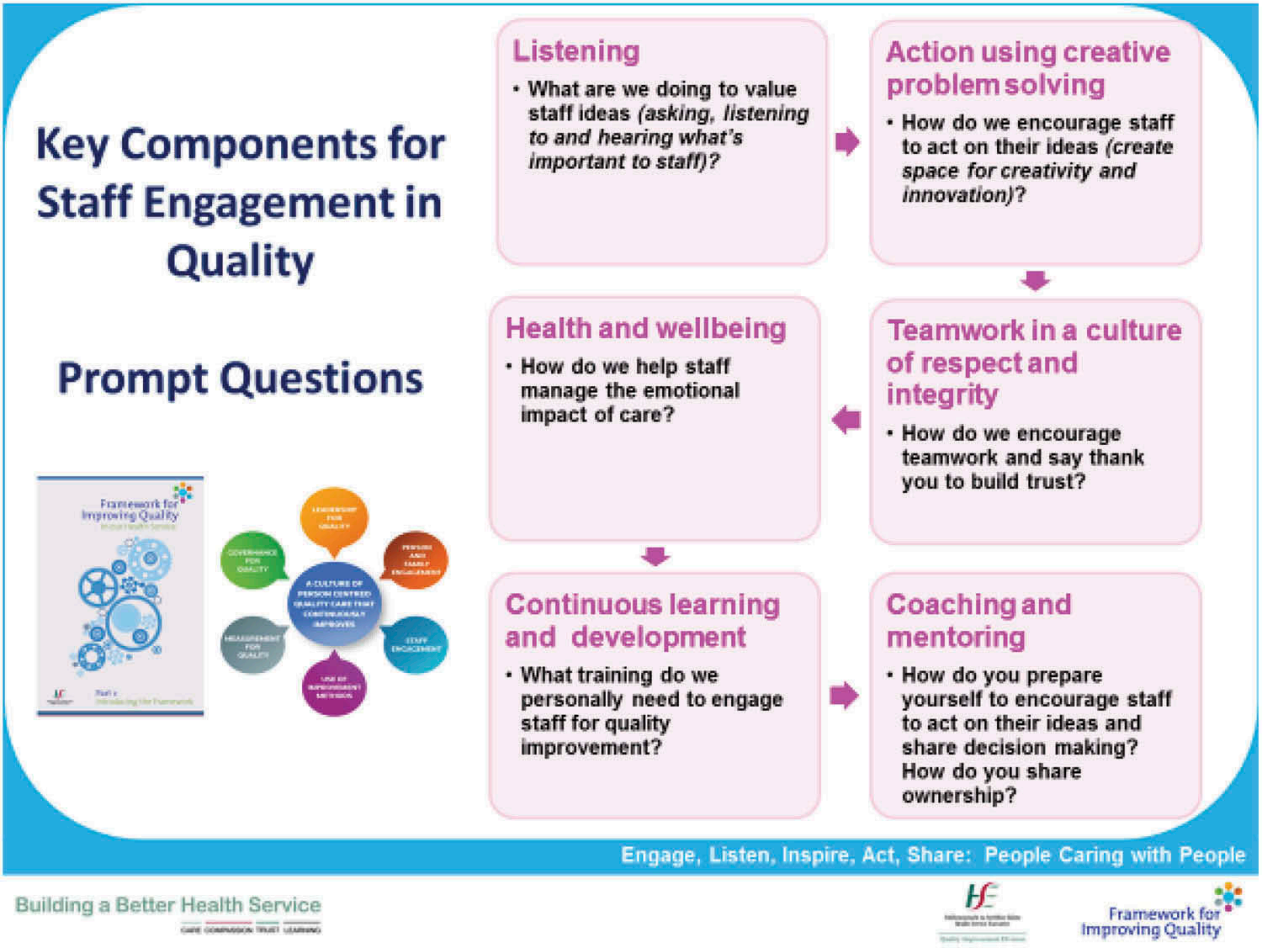
Essential elements for staff engagement in quality.

Questions arising from this presentation included:

“Can it be adapted to an online activity?”.

“Can failure be an outcome?”.

“Can the complex implementation logistics be overcome?”.

Day 2 concluded with ONWARD themed workshops

## Workshop 3A: publishing in CME

Robin Stevenson and Kate Regnier looked at the importance of publishing in CME/CPD, provided writing tips for current and potential authors and posed a series of questions for participants:

- what counts as scholarship?
- what are the first steps toward publication?
- what is the process of reviewer selection?
- how to manage reviewer/author feedback?
- what types of things can be published other than standard manuscripts?
- as authors, what are the top things to consider when submitting a manuscript?
- as reviewers, what kinds of things are being looked for when doing a review?

They referenced a recent report on promoting CME-CPD scholarship/research (June 2017):

http://www.jointaccreditation.org/sites/default/files/2017_Joint_Accreditation_Leadership_Summit_Report.pdf.

The status of CME-CPD publishing was examined and it was noted that there was a paucity of original hypothesis-driven research and that most of the literature dealt with articles written in a review style. A pressing need exists for more case studies (narrative, descriptive reports) and participants were exhorted to reflect on their own work and share challenges, barriers and best practices in the array of publications available.

## Workshop 3B: leadership skills in staff engagement – connect, communicate, engage

Juanita Guidera of the Quality Improvement Division of the Irish Health Service Executive led a workshop to consider the basic leadership principles for engaging staff within a framework for quality improvement and to provide examples of organisational interventions to support connection, communication and engagement.

The main premise for the discussion was that staff are engaged when they feel valued, are emotionally connected, fully involved, enthusiastic and committed to providing a good service and that each person knows that what he or she does and says matters and makes a difference.

The key points for this premise are summarised in Figure 10.

**Figure 11.**
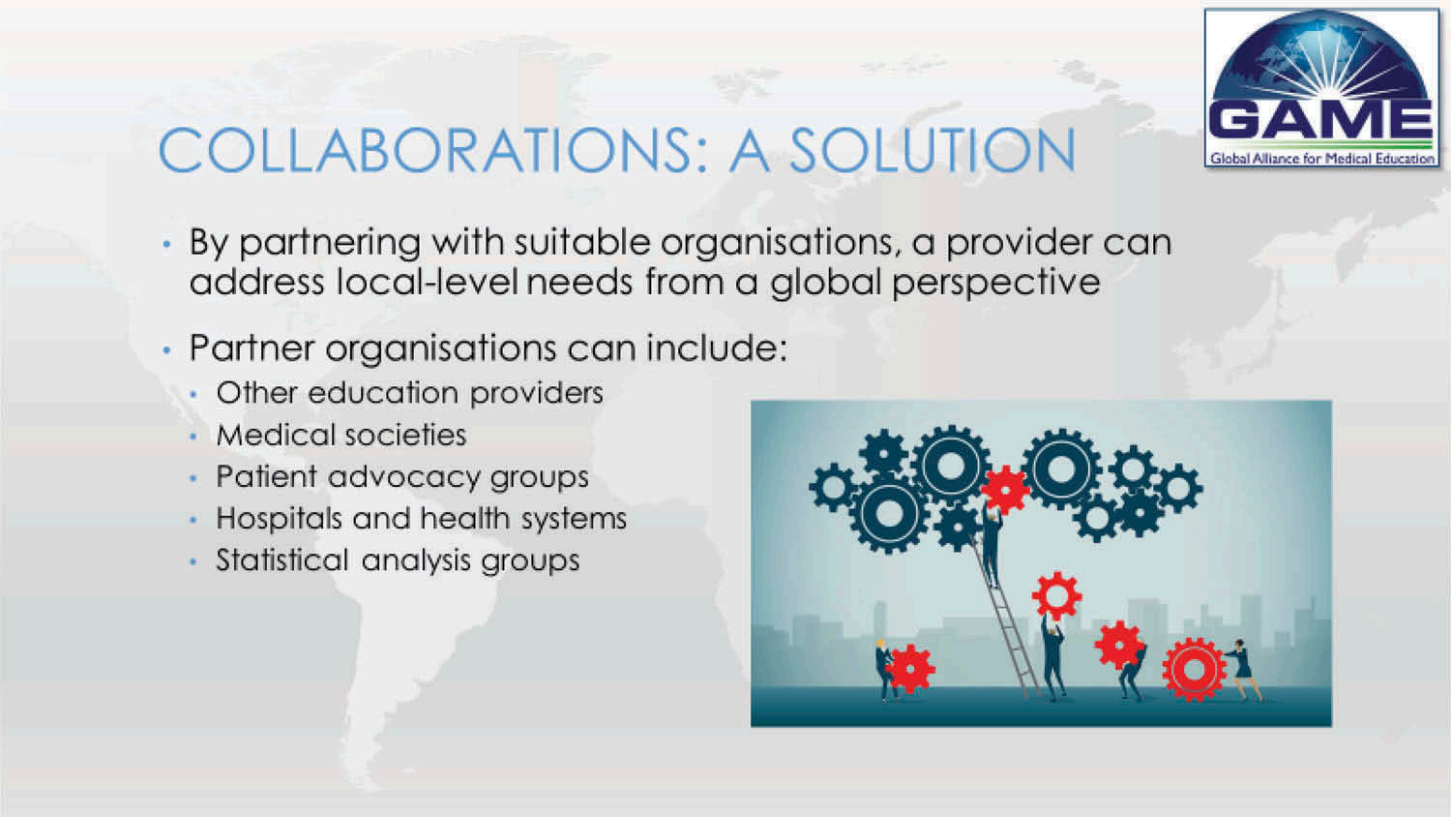
Global partnerships.

Schwartz Rounds (recently implemented in Ireland see: www.theschwartzcenter.org/supporting-caregivers/schwartz-center-rounds/) were cited as an example of a useful mechanism to support staff engagement. These are monthly multidisciplinary meetings attended by staff at all levels that address the emotional impact of being a healthcare professional. The meeting consists of structured, facilitated conversations around a single topic e.g. isolation.

This series was described as the best tool to date for levelling the HCP hierarchy by helping people move away from the control/command management style. The framework improves communication across teams and helps to improve staff well-being, resilience and support which ultimately has an impact on improved patient centred care.

Key take home point: “If you want to take care of someone, take care of yourself first”.

## Workshop 3C: quality collaborations by global organisations

Delegates representing GAME provided a range of perspectives of collaboration on a global scale from the point of view of an educational provider, a financial supporter and a medical society.

The educational provider concluded that there is a global need for education that addresses unique obstacles, such as local codes, guidelines and accreditation bodies, but strategic partnerships can help to overcome challenges. The key to forming these partnerships is a comprehensive analysis of the programme’s aim, identification of organisations that could contribute towards this goal, and how to approach them. Figure 11 illustrates this point.

A global collaboration from a financial supporter’s viewpoint was illustrated by the International Coalition of Hepatology Education Providers that provided 36 programmes in 4 global regions and reached almost 8000 learners. This programme was well received, but also provided insight into region-specific barriers to change that were recognised. Continued support of the programme was a way for educational tailoring to continue to address the most common and pressing educational needs and barriers.

The medical society viewpoint hinged on its current role of providing harmonisation of standards of care and training, dissemination of knowledge, providing relevant content, shaping policy and regulation through partnerships, improving access to best practices, and networking.

## Workshop 3D: future challenges for CME providers in the rapidly evolving CME environment

Diana van Brakel and Toby Borger facilitated a brainstorming session where participants were asked to discuss ways in which CME can be most effective and reinforce best clinical practice in the healthcare setting, identify and address challenges related to the variability in international CME and consider the need for collaboration among CME stakeholders.

Responses indicated that shared best practices to overcome challenges were sought after and that providers still struggle with implementing effective outcomes measurement systems. There was also frustration with the variability in requirements.

Day 3 began with a reflective session on some of the ONWARD presentations from Day 2 with further discussion of the competency-based credit approach being the main point of discussion.

A general conversation session led to a consensus that CME/CPD professionals need to provide their learners with both updated medical skills and communication skills to interact with peers, patients, managers and politicians.

Other points emerging were the importance of team learning, reinforcing what is going right and not just dealing with problems and the need to provide resources for self-directed learning.

The morning continued with a panel discussion on the future of CME mediated by health journalist Jacqui Thornton. Some slightly heated exchanges occurred related to differing opinions on the role of the pharmaceutical industry in CME. Some of these issues have recently been raised elsewhere in this journal and it was refreshing to hear the proponents of differing views sparring verbally rather than in print. The increasing number of workplace-based CME offerings was lauded but some worries were also expressed about the involvement of technology companies in CPD and the potential for “fake” medical education in contrast to the positive benefits of Artificial Intelligence helping with clinical decision-making.

The now traditional “unsession” led by Lawrence Sherman closed the 10th European CME Forum and provided some final points for consideration such as the challenges of compliance rules and regulations in different countries and the hope that more collaboration and sharing of work being done in the European CME arena can be achieved.

Full details of the presentations and support materials may be accessed at the European CME Forum website: http://europeancmeforum.eu

The Twitter stream for the meeting can also be obtained http://europeancmeforum.eu/wp-content/uploads/2016/05/10ECF-Twitter.pdf

The second decade of the Forum will begin in London in November 2018.

R.T. Murray

